# Cholesterol attenuates cytoprotective effects of phosphatidylcholine against bile salts

**DOI:** 10.1038/s41598-017-00476-2

**Published:** 2017-03-22

**Authors:** Yoshito Ikeda, Shin-ya Morita, Tomohiro Terada

**Affiliations:** Department of Pharmacy, Shiga University of Medical Science Hospital, Otsu City, Shiga 520-2192 Japan

## Abstract

Bile salts have potent detergent properties and damaging effects on cell membranes, leading to liver injury. However, the molecular mechanisms for the protection of hepatocytes against bile salts are not fully understood. In this study, we demonstrated that the cytotoxicity of nine human major bile salts to HepG2 cells and primary human hepatocytes was prevented by phosphatidylcholine (PC). In contrast, cholesterol had no direct cytotoxic effects but suppressed the cytoprotective effects of PC. PC reduced the cell-association of bile salt, which was reversed by cholesterol. Light scattering measurements and gel filtration chromatography revealed that cholesterol within bile salt/PC dispersions decreased mixed micelles but increased vesicles, bile salt simple micelles and monomers. These results suggest that cholesterol attenuates the cytoprotective effects of PC against bile salts by facilitating the formation of bile salt simple micelles and monomers. Therefore, biliary PC and cholesterol may play different roles in the pathogenesis of bile salt-induced liver injury.

## Introduction

The biliary lipids consist of a mixture of bile salts, phospholipids and cholesterol. Bile salts serve multiple physiological functions, including generation of bile flow, cholesterol homeostasis, absorption of lipids and vitamins, and antimicrobial defense^1^. In humans, sodium cholate (NaC), sodium deoxycholate (NaDC) and sodium chenodeoxycholate (NaCDC) are major unconjugated bile salts and exclusively conjugated with taurine or glycine in the liver^1^. The proportions of sodium taurocholate (NaTC), sodium glycocholate (NaGC), sodium taurodeoxycholate (NaTDC), sodium glycodeoxycholate (NaGDC), sodium taurochenodeoxycholate (NaTCDC) and sodium glycochenodeoxycholate (NaGCDC) are 13, 25, 6, 15, 14 and 25% of total bile salts in the human gall bladder, respectively^2^. The bile salt concentration exceeds 5 mM in the lumina of bile canaliculi^3^. Bile salts monomers self-associate spontaneously to form simple micelles above their critical micelle concentrations (CMC)^4^. Bile salts have potent detergent properties and deleterious effects upon cell membranes. In bile, the predominant phospholipid is phosphatidylcholine (PC), and cholesterol is unesterified^5–7^. Bile salts are associated with phospholipids and cholesterol in mixed micelles or vesicles, which strongly attenuates the toxicity of bile salts on biliary epithelial cells^8, 9^. Bile salt monomers and simple micelles coexist in dynamic equilibrium with mixed micelles and vesicles, and are responsible for the damaging effects on cell membranes^10, 11^.

ABC transporters expressed on the hepatocyte canalicular membranes are involved in the biliary lipid secretion. ABCB11 participates in most of the bile salt transport from hepatocytes into bile^7, 12, 13^. In the presence of bile salt monomers, ABCB4 mediates the efflux of phospholipids, preferentially PC, into the canalicular lumen^8, 9, 14, 15^. The ABCG5/ABCG8 heterodimer is the main transporter for the secretion of biliary cholesterol^12, 16, 17^.

The cytotoxic effects of bile salts have been shown for hepatocytes and enterocytes^10, 11, 18–21^. The bile salt-induced cell injury is attributed to increased membrane fluidity and permeability after membrane lipid solubilization. The damaging effects of bile salts depend on their degree of hydrophobicity^22^. Previously, we have shown that the expression of phosphatidylethanolamine *N*-methyltransferase in LLC-PK1 cells leads to increased resistance against conjugated bile salts, presumably due to the modifications of phospholipid composition and structure in the apical membranes^22^. Phospholipids, but not cholesterol, provide cytoprotection against bile salt-induced death of Caco-2 cells^10, 11, 18^. However, the roles of phospholipids and cholesterol in the protection against bile salt toxicity on hepatocytes have not been systematically studied, and the underlying mechanism remains poorly understood. In the present study, using the nine major bile salts in humans, we examined the effects of PC and cholesterol on the bile salt toxicity to hepatocytes and on the cell association of bile salts. To elucidate the molecular mechanisms of protection, we also assessed the formation of vesicles, mixed micelles, simple micelles and monomers in the lipid dispersions containing bile salts, PC and cholesterol by light scattering measurements and gel filtration chromatography.

## Results

### Cytotoxicity of bile salts for HepG2 cells

**Figure 1:**
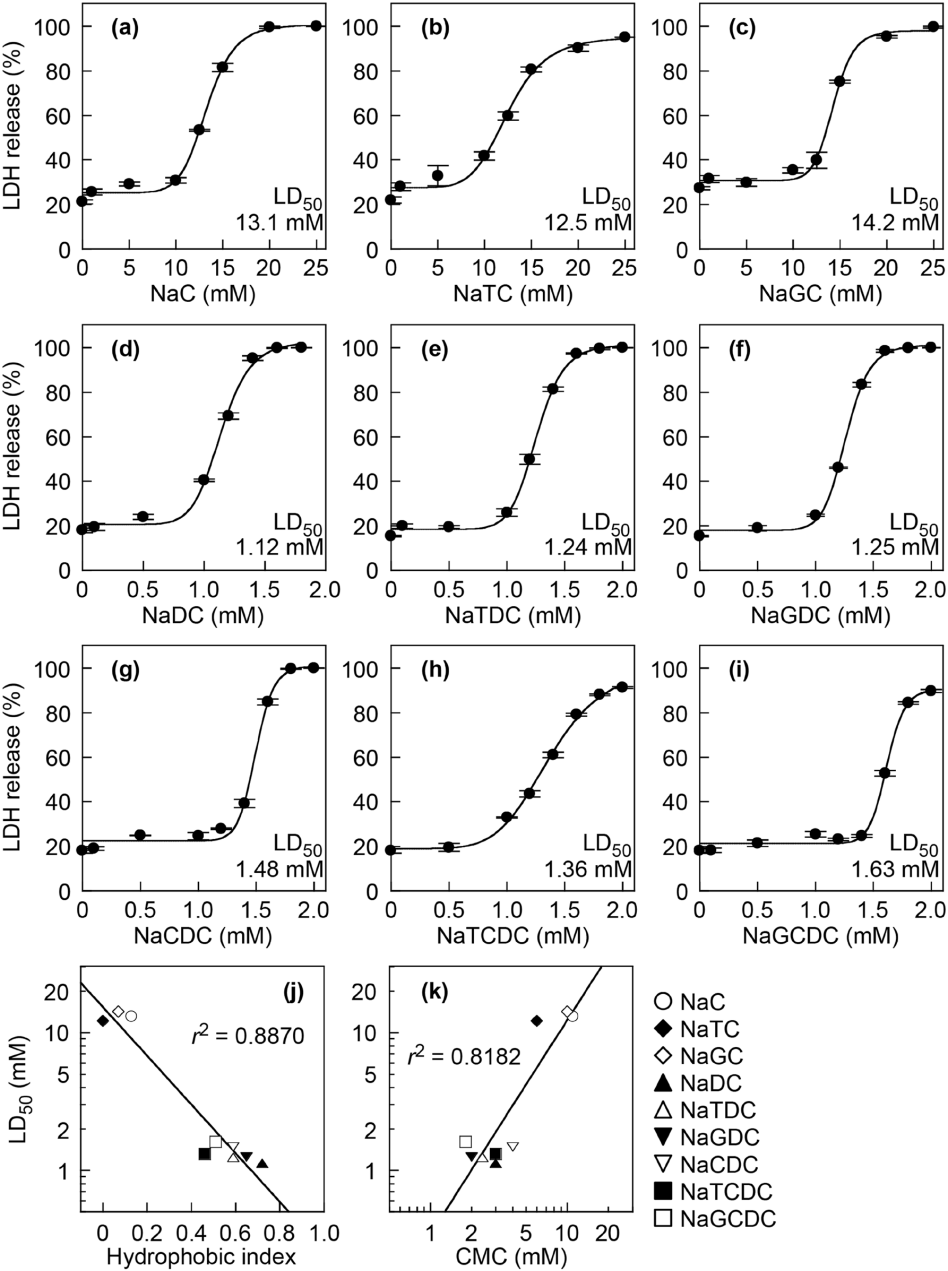
LDH release from HepG2 cells induced by bile salts. HepG2 cells were incubated with NaC (**a**), NaTC (**b**), NaGC (**c**), NaDC (**d**), NaTDC (**e**), NaGDC (**f**), NaCDC (**g**), NaTCDC (**h**) and NaGCDC (**i**) at the indicated concentrations in DMEM containing 0.02% BSA for 30 min at 37 °C. Data represent the mean ± SE (n = 3). The plots of LDH release were fitted to sigmoidal curves. Relationships were observed between the LD_50_ of bile salts for HepG2 cells and hydrophobic index (**j**) or CMC (**k**). LD_50_ values of bile salts were determined by sigmoidal curve fitting of concentration-response data (**a**–**i**). Hydrophobic index of bile salt was reported by Heuman^23^. CMC of bile salt was reported by Roda *et al.* ^4^. The lines were fitted by a single exponential function (**j**, *r* ^2^ = 0.8870) or by a power function (**k**, *r* ^2^ = 0.8182).

To compare the cytotoxicity of the major human bile salts, HepG2 cells were incubated with various concentrations of NaC, NaTC, NaGC, NaDC, NaTDC, NaGDC, NaCDC, NaTCDC or NaGCDC for 30 min, and then the LDH release, an index of irreversible injury or necrosis, was measured. As shown in Fig. 1a–i, all tested bile salts induced LDH release from HepG2 cells in a concentration-dependent manner. NaC and its conjugates were less toxic to HepG2 cells than other bile salts. 50% lethal dose (LD_50_) values of bile salts were determined by sigmoidal curve fitting of concentration-response data (Supplementary Table S1). The bile salt hydrophobic index is derived from relative retention time during high pressure liquid chromatography^23^. The hydrophobic indices of bile salts were strongly inversely correlated with the LD_50_ for HepG2 cells (Fig. 1j). There was also a significant correlation between CMC and LD_50_ of bile salts (Fig. 1k). These results indicated that more hydrophobic bile salts induce more cell damage.

### Effects of PC and cholesterol on bile salt cytotoxicity to HepG2 cells

**Figure 2:**
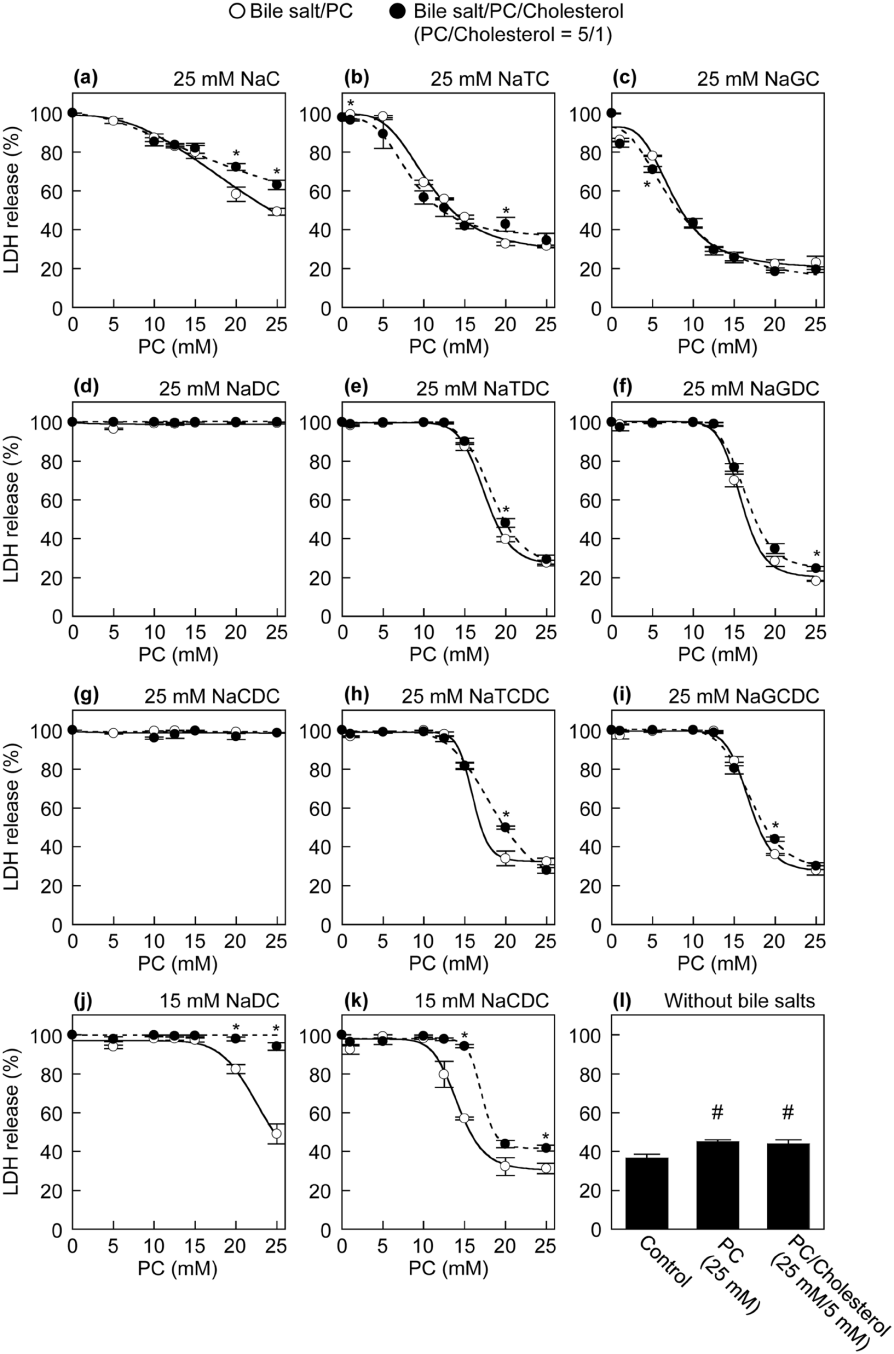
Effects of PC and cholesterol on bile salts cytotoxicity to HepG2 cells assessed by LDH release. HepG2 cells were incubated with 25 mM NaC (**a**), 25 mM NaTC (**b**), 25 mM NaGC (**c**), 25 mM NaDC (**d**), 25 mM NaTDC (**e**), 25 mM NaGDC (**f**), 25 mM NaCDC (**g**), 25 mM NaTCDC (**h**), 25 mM NaGCDC (**i**), 15 mM NaDC (**j**) and 15 mM NaCDC (**k**) in the presence of indicated concentrations of PC (open circles) or PC/cholesterol (5/1) (filled circles) in DMEM containing 0.02% BSA for 30 min at 37 °C. Without bile salts, HepG2 cells were incubated in the absence (control), presence of 25 mM PC, or presence of 25 mM PC and 5 mM cholesterol in DMEM containing 0.02% BSA for 30 min at 37 °C (**l**). Data represent the mean ± SE (n = 3). *P < 0.05, significantly different from bile salt/PC. ^#^ *P* < 0.05, significantly different from the control.

We examined whether PC and cholesterol affect the cytotoxicity induced by various bile salts. To compare the cytotoxicity of various bile salts in the presence of PC and cholesterol, we measured the LDH release from HepG2 cells induced by 25 mM bile salts in the presence of various concentrations of PC and cholesterol. At a concentration of 25 mM, all tested bile salts induced the nearly complete release of LDH from HepG2 cells (Fig. 1a–i). The maximum LDH release was observed in the presence of 2 mM NaDC, NaCDC or their conjugates (Fig. 1d–i), whereas 5 mM NaC, NaTC or NaGC had little or no effect on the cell viability (Fig. 1a–c). The molar ratio of PC/cholesterol is 2.7–14.0 in human bile^24, 25^. In the presence of 25 mM bile salts, except for NaDC and NaCDC, the LDH release from HepG2 cells was progressively decreased with increasing concentration of PC (Fig. 2a–i). Although PC at all tested concentrations did not improve the cytotoxicity of 25 mM NaDC or NaCDC (Fig. 2d,g), high concentrations of PC led to reduced LDH release in the presence of 15 mM NaDC or NaCDC (Fig. 2j,k). Therefore, PC has cytoprotective effects against bile salts. We also determined 50% inhibitory concentration (IC_50_) values of PC for the inhibition of bile salt cytotoxicity (Supplementary Table S2). The cytoprotective effects of PC against unconjugated bile salts were weaker than those against their respective conjugates. Unexpectedly, the LDH release in the presence of bile salts, PC and cholesterol (molar ratio of PC/cholesterol = 5/1) was significantly higher than that in the presence of bile salts and PC (Fig. 2a,b,e,f,h–k). At 15 mM NaDC and NaCDC, prominent differences in the LDH release were observed between PC and PC/cholesterol (Fig. 2j,k). In the cases of 25 mM NaTC, NaTDC, NaTCDC and NaGCDC, the presence of cholesterol significantly increased the LDH release at 20 mM PC but had no influences on the LDH release at 25 mM PC, which may be attributed to almost complete protection against these bile salts by 25 mM PC. Although the treatment with PC or PC/cholesterol (5/1) vesicles resulted in only a slight increase in the LDH release, there was no significant difference in the LDH release between PC and PC/cholesterol vesicles (Fig. 2l). Unfortunately, the cytotoxicity of cholesterol alone cannot be evaluated because cholesterol is highly insoluble in aqueous media without PC or bile salts. From these results, cholesterol has no direct effects on cell viability but attenuates the cytoprotective effects of PC against bile salts.

### Effects of PC and cholesterol on bile salt cytotoxicity to primary human hepatocytes

**Figure 3:**
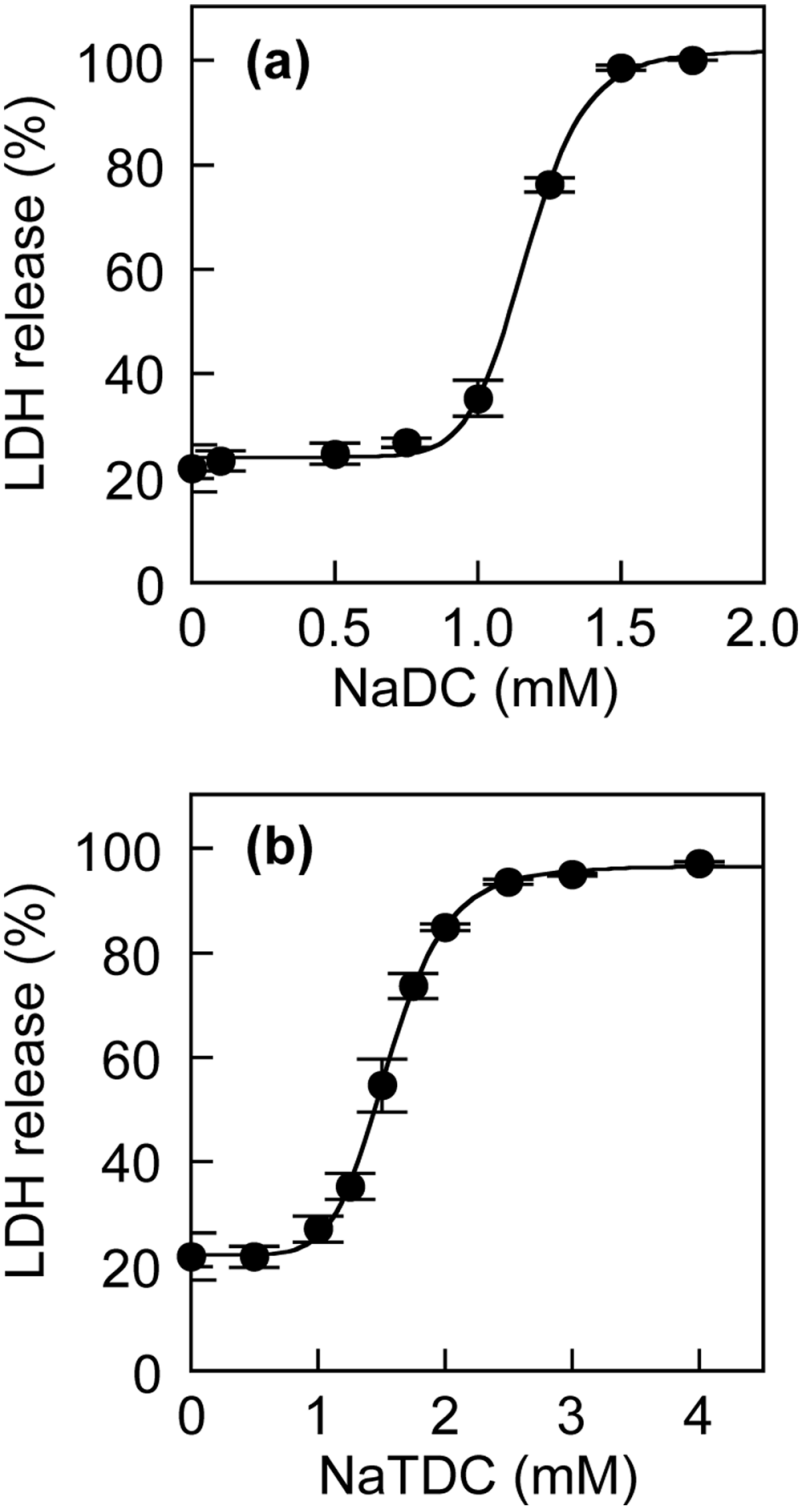
LDH release from primary human hepatocytes induced by bile salts. Primary human hepatocytes were incubated with NaDC (**a**) and NaTDC (**b**) at the indicated concentrations in DMEM containing 0.02% BSA for 30 min at 37 °C. Data represent the mean ± SE (n = 3). The plots of LDH release were fitted to sigmoidal curves.

**Figure 4:**
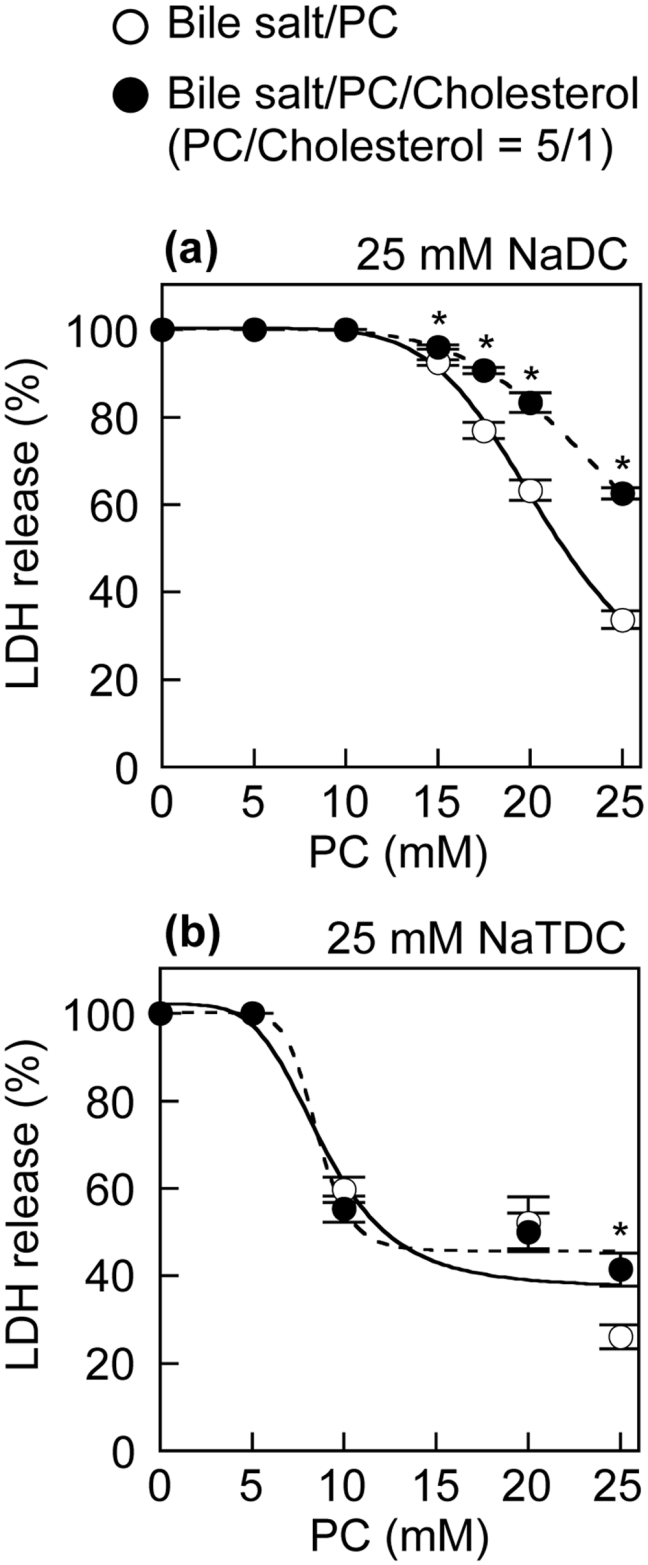
Effects of PC and cholesterol on bile salts cytotoxicity to primary human hepatocytes assessed by LDH release. Primary human hepatocytes were incubated with 25 mM NaDC (**a**) and 25 mM NaTDC (**b**) in the presence of indicated concentrations of PC (open circles) or PC/cholesterol (5/1) (filled circles) in DMEM containing 0.02% BSA for 30 min at 37 °C. Data represent the mean ± SE (n = 3). *P < 0.05, significantly different from bile salt/PC.

Using primary human hepatocytes, we also investigated the effects of PC and cholesterol on the cytotoxicity of bile salts. The LDH release from primary human hepatocytes was concentration-dependently increased by NaDC and NaTDC (Fig. 3a,b), and the LD_50_ values for primary human hepatocytes were very similar to those for HepG2 cells (Supplementary Table S1). In the presence of 25 mM NaDC or NaTDC, an increase in the concentration of PC was accompanied by a decrease in the LDH release from the hepatocytes (Fig. 4a,b). Remarkably, this cytoprotective effect of PC was prevented by the presence of cholesterol (Fig. 4a,b and Supplementary Table S2).

### Effects of PC and cholesterol on cell association of bile salts

**Figure 5:**
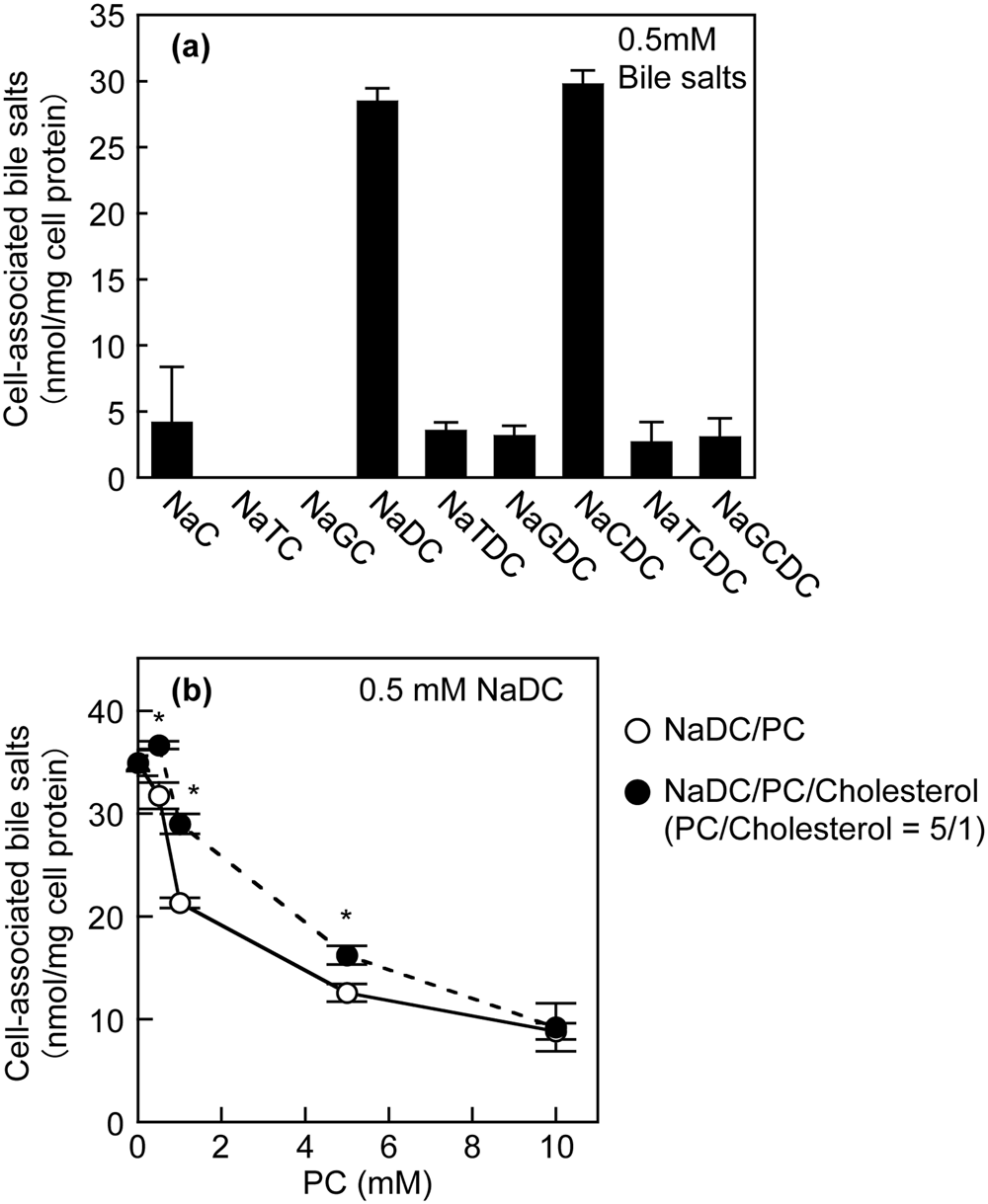
Association of bile salts with cells. (**a**) HepG2 cells were incubated with 0.5 mM NaC, NaTC, NaGC, NaDC, NaTDC, NaGDC, NaCDC, NaTCDC or NaGCDC in DMEM containing 0.02% BSA for 30 min at 37 °C. (**b**) HepG2 cells were incubated with 0.5 mM NaDC in the presence of indicated concentrations of PC (open circles) or PC/cholesterol (5/1) (filled circles) in DMEM containing 0.02% BSA for 30 min at 37 °C. Data represents the mean ± SE (n = 3). *P < 0.05, significantly different from NaDC/PC.

Bile salts have potent detergent properties and damage cells by affecting the integrity of cellular membranes. We assessed the association of bile salts with HepG2 cells. The sodium-taurocholate cotransporting polypeptide (NTCP) and organic anion-transporting polypeptide (OATP), transporters for bile salt uptake, are not expressed in HepG2 cells^26, 27^. To evaluate the cell association of various bile salts, HepG2 cells were incubated with 0.5 mM bile salts because all tested bile salts at a concentration of 0.5 mM did not affect the cell viability (Fig. 1a–i). The amounts of cell-associated bile salts cannot be determined at concentrations that induce cell death. As shown in Fig. 5a, the amounts of cell-associated NaDC and NaCDC were remarkably higher than those of other bile salts. Next, we examined the effects of PC and cholesterol on the cell-association of NaDC. The cell-association of NaDC reduced with increasing PC concentration (Fig. 5b). On the other hand, the inclusion of cholesterol within NaDC/PC dispersions resulted in significantly higher cell-association of NaDC, indicating cholesterol reverses the suppressive effects of PC on the cell-association of NaDC. Thus, the effects of PC and cholesterol on NaDC cytotoxicity may be attributed to the alteration of cell-association.

### Distribution of bile salts between vesicles, mixed micelles, simple micelles and monomers

**Figure 6:**
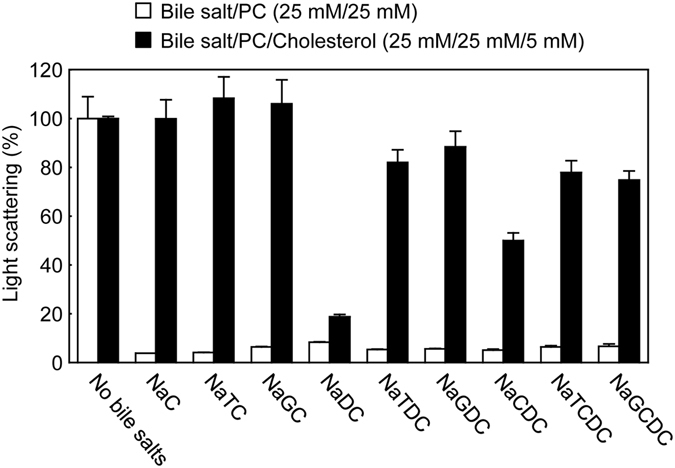
Light scattering intensity of bile salt/PC and bile salt/PC/cholesterol dispersions. Light scattering intensity of 25 mM bile salt/25 mM PC (open bars) or 25 mM bile salt/25 mM PC/5 mM cholesterol (filled bars) dispersions was measured at 37 °C. The light scattering intensity of each dispersion in the absence of bile salts was taken as 100%. Values represent the mean ± SD (n = 3). The absence of an error bar signifies an SD value smaller than the graphic symbol.

It has been suggested that the formation of bile salt/PC mixed micelles and vesicles inactivates the membrane lytic action of bile salts, whereas bile salt monomers and simple micelles exert damaging effects on the membrane bilayer^10, 11^. Mixed micelle formation in the lipid dispersions was estimated by measuring light scattering intensity. Vesicles are large particles causing strong light scattering, whereas micelles are small particles and weakly scatter light. As shown in Fig. 6, the dispersions containing bile salts and PC exhibited very low light scattering intensity compared with control PC vesicles, probably due to the formation of bile salt/PC mixed micelles. In contrast, the light scattering of bile salt/PC/cholesterol dispersions was similar or somewhat lower than that of PC/cholesterol vesicles, suggesting that cholesterol prevents the formation of mixed micelles.

**Figure 7:**
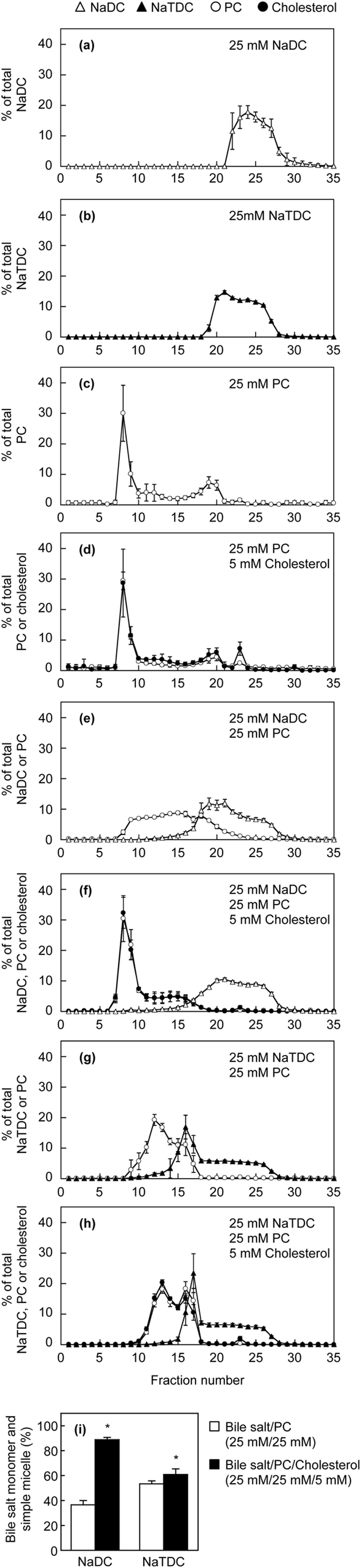
Gel filtration of lipid dispersions. 25 mM NaDC (**a**), 25 mM NaTDC (**b**), 25 mM PC (**c**), 25 mM PC/5 mM cholesterol (**d**), 25 mM NaDC/25 mM PC (**e**), 25 mM NaDC/25 mM PC/5 mM cholesterol (**f**), 25 mM NaTDC/25 mM PC (**g**) and 25 mM NaTDC/25 mM PC/5 mM cholesterol (**h**) dispersions were separated by gel filtration chromatography on Superose 6 Increase at 37 °C. The percentages of NaDC (open triangles), NaTDC (filled triangles), PC (open circles) and cholesterol (filled circles) in each fraction were measured by enzymatic assays. Data represent the mean ± SD (**a**–**f**, n = 3; **g**,**h**, n = 4). The absence of an error bar signifies an SD value smaller than the graphic symbol. The gel filtration profiles (**e**–**h**) were used to determine the percentages of bile salt monomers and simple micelles in 25 mM bile salt/25 mM PC (open bars) and 25 mM bile salt/25 mM PC/5 mM cholesterol (filled bars) (**i**). Bile salt molecules in fractions 23–35 (**e**) or 18–35 (**f**–**h**) existed exclusively as monomers or simple micelles. Each bar represents the mean ± SD (NaDC, n = 3; NaTDC, n = 4). *P < 0.05, significantly different from bile salt/PC (25 mM/25 mM).

Using gel filtration chromatography, we also determined the distribution of bile salts between vesicles, mixed micelles, simple micelles and monomers. The lipid particles, vesicles and micelles, are separated based on their sizes by gel filtration chromatography. Figure 7a–h display the elution profiles of the lipid dispersions. NaDC simple micelles and monomers were eluted in fractions 22–30 (Fig. 7a). The simple micellar peak of NaTDC was eluted slightly faster than that of NaDC (Fig. 7a,b). The peaks of PC and PC/cholesterol vesicles were observed in the void volume (fraction 8) (Fig. 7c,d). The elution behaviors of PC and cholesterol were extremely similar (Fig. 7d), indicating cholesterol was uniformly distributed in PC bilayers. In the elution profile of NaDC/PC dispersions, the vesicular and simple micellar peaks were markedly reduced, and NaDC and PC were broadly distributed and coexisted in the fractions between vesicular and simple micellar peaks (Fig. 7e), suggesting the formation of NaDC/PC mixed micelles. PC was recovered in fractions 8–22, indicating fractions 23–35 corresponded to the simple micelles and monomers of NaDC. On the other hand, in the profile of NaDC/PC/cholesterol dispersions, the large vesicular peak remained, and NaDC hardly coexisted with PC and cholesterol in the same fractions (Fig. 7f), suggesting only small amounts of mixed micelles. NaDC molecules in fractions 18–35 were present as simple micelles and monomers. Furthermore, PC and cholesterol showed the same distribution patterns despite the presence of NaDC. The percentage of NaDC simple micelles and monomers was strikingly higher in NaDC/PC/cholesterol dispersions than in NaDC/PC dispersions (Fig. 7i). In both cases of NaTDC/PC and NaTDC/PC/cholesterol dispersions, the peaks of PC became later and lower, but NaTDC simple micelles and monomers were found in fractions 18–35 (Fig. 7g,h). Even in the presence of NaTDC, cholesterol was distributed similarly to PC. NaTDC/PC/cholesterol dispersions contained more simple micelles and monomers than NaTDC/PC dispersions (Fig. 7i). Taken together, these data suggest that cholesterol in the lipid dispersions promotes cell death by increasing the proportion of bile salt simple micelles and monomers but not mixed micelles.

## Discussion

Bile salts, phospholipids and cholesterol are the major lipid components of bile. In bile, mixed micelles and vesicles, both of which are composed of bile salts, cholesterol and phospholipids, coexist in dynamic equilibrium with bile salt monomers and simple micelles. Cholesterol is almost entirely insoluble in water, and the solubility of cholesterol is 3.7 × 10^−8^ M^28^. The simple micelles are small (~3 nm in diameter) and solubilize very small quantities of biliary cholesterol^6^. For example, NaDC or NaTC micelles solubilize cholesterol at a molar ratio of approximately 1/20 or 1/100, respectively^29^. At low phospholipid/bile salt ratios (<1/4), phospholipid molecules are intercalated in mixed micelles^5^. Mixed micelles are quasispherical (4–8 nm in diameter)^6^. The cholesterol solubilizing capacity of mixed micelles (1 cholesterol per 3 phospholipid and >12 bile salt molecules) is higher than that of simple bile salt micelles^5^. Vesicles are spherical structures (40–100 nm in diameter) and poor in bile salts (phospholipid/bile salt ratio of >1/4)^5, 6^. Multilamellar vesicles (>500 nm in diameter) may be derived from fusion of unilamellar vesicles^6^. Both unilamellar and multilamellar vesicles are seen in human bile^3, 5, 6^. Some bile salt molecules are intercalated among phospholipid molecules in the bilayer structures and have an influence on the shape of vesicles^5^. Compared with mixed micelles, vesicles are much more efficient cholesterol carriers requiring only 1 phospholipid molecule to solubilize 1 cholesterol molecule^5^. Thus, mixed micelles are inefficient cholesterol solubilizers. Biliary cholesterol solubilization is dependent not only on the concentration of cholesterol itself but also on the phospholipid/bile salt ratio. Meanwhile, the dissolution rate of vesicles by various bile salts is dependent on the cholesterol/phospholipid ratio^30^. Vesicles with a low cholesterol/phospholipid ratio are rapidly dissolved, whereas an increase in the cholesterol/phospholipid ratio makes them more resistant to the solubilizing action of bile salts. Noteworthy, the inclusion of cholesterol in PC vesicles reduces the binding affinity of taurine-conjugated bile salts^31^. Mello-Vieira *et al.* have recently demonstrated that the fluorescent-labeled deoxycholic acid partitions with higher affinity to the liquid disordered domains than to the cholesterol-enriched ordered domains on lipid vesicles, and that the unlabeled bile acids have a superficial location upon interaction with the lipid membranes^32^. In the present study, we prepared lipid dispersions containing bile salts, PC and cholesterol. According to the equilibrium bile salt/PC/cholesterol ternary phase diagram constructed by Wang and Carrey^6, 33^, bile salt/PC and bile salt/PC/cholesterol (PC/cholesterol = 5/1) lipid dispersions used in our experiments may be located in the one-phase micellar zone or the two-phase zone containing micelles and vesicles, in which there are no cholesterol crystals. By light scattering measurements and gel filtration chromatography, we demonstrated that cholesterol suppressed the formation of mixed micelles but facilitated that of simple micelles and monomers, and that PC and cholesterol were similarly distributed between vesicles and mixed micelles (Figs 6 and 7). It is conceivable that cholesterol associates strongly and preferentially with PC, prevents the interaction of bile salts with PC, and thermodynamically stabilizes the vesicle structures, which leads to decreased formation of mixed micelles and to increased amounts of bile salt simple micelles and monomers.

The bile salt-induced liver damage is suggested to depend on the total amount and the hydrophobic-hydrophilic balance of bile salt species^22^. In the present study, we used HepG2 cells and primary human hepatocytes for evaluating the bile salt toxicity. Our data showed correlations of bile salt cytotoxicity to HepG2 cells with hydrophobic index and CMC (Fig. 1j,k), indicating that hydrophobic bile salts are more cytotoxic than hydrophilic bile salts. The differences in the relative cytotoxicity among bile salts are likely due to both amounts of cell-associated bile salts and direct toxic effects of bile salts including the abilities to disrupt cell membranes and to alter intracellular signaling. The association of bile salts with HepG2 cells probably occurs by passive diffusion, because HepG2 cells do not express NTCP or OATP^26, 27^. In the liver, NTCP and OATP are localized in the basolateral membranes of hepatocytes and mediate the uptake of bile salts from sinusoidal blood^1, 34^, whereas the biliary bile salts are accessible only to the hepatocyte canalicular membranes but not to the basolateral membranes. Thus, NTCP and OATP on the basolateral membranes may not play major roles in the cytotoxicity of bile salts in bile canalicular lumina to hepatocytes *in vivo*. Although primary human hepatocytes may express bile salt transporters, the cytotoxicity of bile salts was similar between primary human hepatocytes and HepG2 cells (Figs 1 and 3 and Supplementary Table S1).

In addition, monomeric and simple micellar bile salts, rather than mixed micellar bile salts, may exert detergent effects on membrane bilayers. Both PC and cholesterol have been shown to reduce NaTC-induced lysis of human erythrocytes^35^. However, the lipid composition and relative vulnerability of erythrocyte membranes are very different from those of hepatocyte membranes^18, 36^. The incubation of erythrocytes with a NaTC/cholesterol solution leads to a marked increase in the membrane cholesterol content and to a concomitant reduction in the membrane PC content^18^. The high cholesterol/PC ratio in the membranes may protect the erythrocytes against bile salts, because the erythrocyte membranes with low fluidity are less readily lysed than those with higher fluidity^37^. Previous studies have also shown that the incorporation of PC in NaTC micelles profoundly inhibits the LDH release from Caco-2 cells induced by NaTC^10, 11^. Velardi *et al.* have demonstrated that cholesterol alone has no protective effects on Caco-2 cells but increases cytotoxicity in combination with NaTC and PC, which correlates with a cholesterol-induced shift of PC from micelles to vesicles in model bile containing NaTC^18^. It has been proposed that the bile salt molecules bind and penetrate into the phospholipid bilayers, and the saturation of the phospholipid bilayers by the bile salt molecules then leads to the solubilization of bilayers into bile salt/phospholipid mixed micelles^38^. In addition, bile salts are cytotoxic via intracellular processes, such as mitochondria-mediated toxicity^39^. Therefore, the cell-association of bile salt may be a critical step for cell death. In this study, we demonstrated that PC decreased the bile salt toxicity to HepG2 cells and primary human hepatocytes, which was attenuated by cholesterol (Figs 2 and 4). Furthermore, PC prevented the cell-association of NaDC, but this effect of PC is reversed by cholesterol (Fig. 5b). From these results, we suggest that the monomeric and simple micellar bile salts, rather than bile salts in mixed micelles, predominantly associate with cell membranes and subsequently induce cell death, which is regulated by phospholipids and cholesterol in lipid dispersions. The weak cytoprotective effects of PC against NaDC and NaCDC (Fig. 2d,g) are presumably due to the high cell-association of NaDC and NaCDC (Fig. 5a).

The biliary concentrations of bile salts, phospholipids and cholesterol differ between individuals and are mainly determined by specific transporters, ABCB11 for bile salts, ABCB4 for phospholipids and ABCG5/ABCG8 for cholesterol. *Abcb4* knockout mice display normal secretion of bile salts, but have an almost complete absence of phospholipids and cholesterol from bile, which results in liver disease characterized by severe necrotic damage of hepatocytes^40, 41^. Human *ABCB4* mutations result in progressive familial intrahepatic cholestasis type 3, intrahepatic cholestasis of pregnancy, low phospholipid-associated cholelithiasis, primary biliary cirrhosis, cholangiocarcinoma and hepatocellular carcinoma^8, 9, 42, 43^. In addition, bile duct injury occurring after liver transplantation correlates with the formation of toxic bile with a high bile salt/phospholipid ratio^44^. The secretion of cholesterol via ABCG5/ABCG8 requires phospholipids secreted by ABCB4^45^. An elevation in biliary cholesterol concentration results in supersaturation of cholesterol in bile, subsequent precipitation of cholesterol crystals and cholesterol gallstone formation^7^. Farnesoid X receptor regulates the expression of ABCB11 and ABCB4 in the hepatocyte canalicular membrane, while liver X receptor regulates ABCG5/ABCG8 expression^7^. Farnesoid X receptor functions as a bile salt receptor and regulates the transcription of numerous genes involved in maintaining cholesterol and bile salt homoeostasis. Additionally, miR-33 controls the hepatic expression of Abcb11 and Abcg5/Abcg8 in mice and increases the relative amount of cholesterol in bile^46^. The regulation of expression of ABCG5/ABCG8, in addition to ABCB4 and ABCB11, is likely to be crucial for the protection of hepatocytes against bile salts. Yoshikado *et al.* have suggested that itraconazole-induced cholestasis involves the inhibition of ABCB4-mediated biliary phospholipid secretion^47^. Chronic legume feeding leads to increases in the biliary cholesterol secretion in humans^48, 49^. Bezafibrate and fenofibrate have been shown to increase the biliary cholesterol secretion^50^. Most recently, Schonewille *et al.* have reported that rosuvastatin, atorvastatin and lovastatin enhance the biliary cholesterol secretion^51^. In contrast, pravastatin induces a decrease in the biliary cholesterol secretion in healthy volunteers^52^. Bile containing normal levels of bile salts, PC and cholesterol does not lead to hepatocellular damage. The hepatobiliary system maintains a delicate balance between bile salts, PC and cholesterol. Our results demonstrated that, under some but not all conditions, cholesterol prevented the cellular protection by PC against bile salts (Figs 2 and 4). Therefore, by pathological condition, food consumption or drug administration, a moderate decrease in biliary PC and/or a moderate increase in biliary cholesterol may cause hepatocellular injury induced by bile salts.

In addition, bile salts have been reported to activate transmembrane receptors such as G-protein-coupled bile acid receptor 1 (GPBAR1, also known as TGR5), sphingosine-1-phosphate receptor 2 (S1PR2) and α5β1 integrins^34, 53, 54^. TGR5 is widely distributed throughout the body and is expressed by Kupffer cells, sinusoidal endothelial cells and cholangiocytes in the mouse liver^34^. The order of potency for activation of TGR5 by bile salts is lithocholate ≥ deoxycholate > chenodeoxycholate > cholate, with taurine conjugates being more potent than glycine conjugates^34^. The activation of TGR5 stimulates cholangiocyte proliferation and prevents death receptor-mediated apoptosis^34^. It has been shown that TGR5 protects the liver against bile acid overload after partial hepatectomy^55^. S1PR2 is highly expressed in hepatocytes and cholangiocytes and is activated by conjugated bile salts but not unconjugated bile salts^54^. The conjugated bile salts activate S1PR2, increase sphingosine kinase 2 expression and nuclear sphingosine-1-phosphate, and regulate lipid and sterol metabolism in the liver^54^. Conjugated bile salts activate the extracellular regulated kinase 1/2 and protein kinase B signaling pathways primarily through S1PR2 in hepatocytes^56^. Tauroursodeoxycholate, but not other conjugated bile salts, activates α5β1 integrins in hepatocytes^53^. It is possible that biliary PC and cholesterol regulate the signaling through TGR5, S1PR2 or α5β1 integrins by altering the amounts of monomeric and simple micellar bile salts.

In conclusion, PC protects hepatocytes from the cytotoxicity of bile salts by decreasing the cell-association of bile salts. In contrast, cholesterol reverses the protective effects of PC against bile salt cytotoxicity, which can be accounted for by increased formation of bile salt simple micelles and monomers. Therefore, biliary PC and cholesterol may differently regulate the progression of liver injury induced by bile salts.

## Methods

### Materials

NaC and NaDC were obtained from Wako Pure Chemical Industries (Osaka, Japan). NaTC, NaGC, NaTDC, egg yolk PC, cholesterol and 4-aminoantipyrine were purchased from Nacalai Tesque (Kyoto, Japan). NaGDC, NaCDC, NaTCDC and NaGCDC were purchased from Sigma-Aldrich (St. Louis, MO, USA). Glycerophospholipid-specific phospholipase D from *Streptomyces* sp. was purchased from Asahi Kasei Pharma (Tokyo, Japan). Recombinant cholesterol oxidase from *Nocardia* sp. and peroxidase from horseradish roots was obtained from Oriental Yeast (Suita, Osaka, Japan). *N*-Ethyl-*N*-(2-hydroxy-3-sulfopropyl)-3,5-dimethoxyaniline sodium salt (DAOS) was purchased from Dojindo Laboratories (Kumamoto, Japan). All other chemicals used were of the highest reagent grade available.

### Preparation of lipid dispersions

Egg yolk PC is similar to PC in human bile^5, 10, 24^. The thin films of egg yolk PC and cholesterol obtained by evaporating the lipid chloroform solution were hydrated with phenol red-free Dulbecco’s modified Eagle’s medium (DMEM) containing 0.02% bovine serum albumin (BSA), and were subsequently ultrasonicated (model UR-20P, Tomy Seiko, Tokyo, Japan) for 1 min on ice. Then, bile salts were added to the lipid dispersions.

### Cell cultures

HepG2 cells, a human hepatoma cell line, were purchased from Riken BioResource Center (Tsukuba, Japan). HepG2 cells were grown in a humidified incubator (5% CO_2_) at 37 °C in DMEM supplemented with 10% heat-inactivated fetal bovine serum (FBS). HepG2 cells were subcultured in 24-well plates at a density of 4.0 × 10^4^ cells or in 6-well plates at a density of 1.0 × 10^6^ cells and incubated for 48 h.

The cryopreserved primary human hepatocytes isolated from two Caucasians (82-year-old man and 51-year-old woman) were purchased from KaLy-Cell (Plobsheim, France). KaLy-Cell has the ability by the French Ministry of Research to prepare and store products from human origin for research purposes and to export human samples. The hepatocytes were thawed in KLC-Thawing Medium (KaLy-Cell), washed and resuspended in KLC-Seeding Medium (KaLy-Cell). The hepatocytes were seeded in 48-well plates coated with type I collagen at a density of 1.0 × 10^5^ cells in a humidified incubator (5% CO_2_) at 37 °C and incubated for 48 h.

### LDH release assay

After incubation for 48 h, HepG2 cells in 24-well plates or primary human hepatocytes in 48-well plates were washed with phenol red-free DMEM containing 0.02% BSA. The cells were incubated for 30 min with phenol red-free DMEM containing 0.02% BSA in the absence or presence of various concentrations of bile salts, PC and cholesterol. The cells were then dissolved in 1% Triton X-100. Cytotoxicity of bile salts was estimated by measuring the LDH activity in the media and total cells using a CytoTox-ONE homogeneous membrane integrity assay kit (Promega, Madison, WI, USA)^22, 57^. %LDH release = LDH activity in media/(LDH activity in media + LDH activity in total cells) × 100%. The plots of LDH release induced by bile salts were fitted to a sigmoidal curve.

### Assessment of cell-associated bile salts

After incubation for 48 h, HepG2 cells in 6-well plates were washed with phenol red-free DMEM containing 0.02% BSA. The cells were incubated for 30 min with phenol red-free DMEM containing 0.02% BSA and 0.5 mM bile salts in the absence or presence of various concentrations of PC and cholesterol. The cells were chilled on ice, washed twice with cold HEPES buffer (137 mM NaCl, 5.4 mM KCl, 0.6 mM MgCl_2_, 1.1 mM CaCl_2_, 6.1 mM D-glucose and 10 mM HEPES; pH 7.4), dissolved in 1% Triton X-100 and sonicated. The amount of bile salts was measured using an enzymatic assay kit (Wako Pure Chemical Industries). The protein content was determined using a BCA protein assay kit (Thermo Scientific, Waltham, MA). The amount of cell-associated bile salts was calculated by subtracting that of endogenous bile salts in HepG2 cells.

### Light scattering measurements

Light scattering measurements were performed as previously described^14^. The right-angle light scattering of the lipid dispersions was measured by a spectrofluorometer (F-4500, Hitachi High-Technologies, Tokyo, Japan) at 37 °C using excitation and emission wavelengths of 400 nm.

### Gel filtration chromatography

The lipid dispersions (500 µl) were loaded on a Superose 6 Increase 10/300 GL column (GE Healthcare, Buckinghamsihre, UK) and eluted at 37 °C with HEPES buffer (137 mM NaCl, 5.4 mM KCl, 6.1 mM D-glucose and 10 mM HEPES; pH 7.4) at a rate of 1.5 ml/min using a BioLogic DuoFlow system (Bio-Rad Laboratories, Hercules, CA). Fractions (1.0 ml each) were collected, and the amount of bile salts in each fraction was determined using an enzymatic assay kit (Wako Pure Chemical Industries). The amounts of PC and cholesterol in each fraction were measured by enzymatic assays.

### Enzymatic measurements of PC and cholesterol

The amounts of PC and cholesterol were quantified by enzymatic methods as previously described^58–60^. The enzymatic measurement of PC was performed using a one-reagent system. Briefly, Reagent C contained 4 units/ml glycerophospholipid-specific phospholipase D, 4 units/ml choline oxidase, 5 units/ml peroxidase, 1 mM 4-aminoantipyrine, 1 mM DAOS, 0.2% Triton X-100, 1.1 mM CaCl_2_, 50 mM NaCl and 50 mM Tris-HCl (pH 7.4). Sample (10 µl) was added to 200 µl of Reagent C, and incubated at 37 °C for 20 min. Absorption was measured at 595 nm using a microplate reader (Infinite M200, Tecan, Männedorf, Switzerland).

The enzymatic measurement of cholesterol was performed using a one-reagent system. Briefly, Reagent CH contained 1 unit/ml cholesterol oxidase, 2.5 units/ml peroxidase, 1 mM 4-aminoantipyrine, 1 mM DAOS, 0.2% Triton X-100, 50 mM NaCl and 50 mM Tris-HCl (pH 7.4). Sample (10 µl) was added to 90 µl of Reagent CH and incubated at room temperature for 30 min. Absorption was measured at 595 nm using a Tecan Infinite M200 microplate reader.

### Statistical analysis

The statistical significance of differences between mean values was determined using the unpaired Student’s *t*-test. Multiple comparisons were performed using the Bonferroni test following ANOVA. Differences were considered significant at *P* < 0.05.

## Electronic supplementary material

Supplementary Tables

## References

[1] Hofmann AF, Hagey LR (2008). Bile acids: chemistry, pathochemistry, biology, pathobiology, and therapeutics. Cell Mol Life Sci.

[2] Eckhardt ER, van de Heijning BJ, van Erpecum KJ, Renooij W, VanBerge-Henegouwen GP (1998). Quantitation of cholesterol-carrying particles in human gallbladder bile. J Lipid Res.

[3] Crawford AR (1997). Hepatic secretion of phospholipid vesicles in the mouse critically depends on mdr2 or MDR3 P-glycoprotein expression. Visualization by electron microscopy. J Clin Invest.

[4] Roda A, Hofmann AF, Mysels KJ (1983). The influence of bile salt structure on self-association in aqueous solutions. J Biol Chem.

[5] Gilat T, Somjen GJ (1996). Phospholipid vesicles and other cholesterol carriers in bile. Biochim Biophys Acta.

[6] Wang, D.Q., Cohen, D.E. & Carey, M.C. Biliary lipids and cholesterol gallstone disease. *J Lipid Res* **50** Suppl, S406–S411 (2009). 10.1194/jlr.R800075-JLR200 PMC2674701 19017613

[7] van Erpecum KJ (2005). Biliary lipids, water and cholesterol gallstones. Biol Cell.

[8] Oude Elferink RP, Paulusma CC (2007). Function and pathophysiological importance of ABCB4 (MDR3 P-glycoprotein). Pflugers Arch.

[9] Morita SY, Terada T (2014). Molecular mechanisms for biliary phospholipid and drug efflux mediated by ABCB4 and bile salts. Biomed Res Int.

[10] Moschetta A (2001). Hydrophilic bile salts enhance differential distribution of sphingomyelin and phosphatidylcholine between micellar and vesicular phases: potential implications for their effects *in vivo*. J Hepatol.

[11] Moschetta A (2000). Sphingomyelin exhibits greatly enhanced protection compared with egg yolk phosphatidylcholine against detergent bile salts. J Lipid Res.

[12] Small DM (2003). Role of ABC transporters in secretion of cholesterol from liver into bile. Proc Natl Acad Sci USA.

[13] Arrese M, Ananthanarayanan M (2004). The bile salt export pump: molecular properties, function and regulation. Pflugers Arch.

[14] Morita SY (2007). Bile salt-dependent efflux of cellular phospholipids mediated by ATP binding cassette protein B4. Hepatology.

[15] Morita SY (2013). Bile salt-stimulated phospholipid efflux mediated by ABCB4 localized in nonraft membranes. J Lipid Res.

[16] Yu L (2002). Overexpression of ABCG5 and ABCG8 promotes biliary cholesterol secretion and reduces fractional absorption of dietary cholesterol. J Clin Invest.

[17] Yu L (2002). Disruption of Abcg5 and Abcg8 in mice reveals their crucial role in biliary cholesterol secretion. Proc Natl Acad Sci USA.

[18] Velardi AL (1991). Cell type-dependent effect of phospholipid and cholesterol on bile salt cytotoxicity. Gastroenterology.

[19] Martinez-Diez MC, Serrano MA, Monte MJ, Marin JJ (2000). Comparison of the effects of bile acids on cell viability and DNA synthesis by rat hepatocytes in primary culture. Biochim Biophys Acta.

[20] Akare S, Martinez JD (2005). Bile acid induces hydrophobicity-dependent membrane alterations. Biochim Biophys Acta.

[21] Jean-Louis S (2006). Deoxycholic acid induces intracellular signaling through membrane perturbations. J Biol Chem.

[22] Morita SY (2011). Effects of phosphatidylethanolamine N-methyltransferase on phospholipid composition, microvillus formation and bile salt resistance in LLC-PK1 cells. FEBS J.

[23] Heuman DM (1989). Quantitative estimation of the hydrophilic-hydrophobic balance of mixed bile salt solutions. J Lipid Res.

[24] Hay DW, Cahalane MJ, Timofeyeva N, Carey MC (1993). Molecular species of lecithins in human gallbladder bile. J Lipid Res.

[25] Gauss A (2013). Biliary phosphatidylcholine and lysophosphatidylcholine profiles in sclerosing cholangitis. World J Gastroenterol.

[26] Okuyama-Dobashi K (2015). Hepatitis B virus efficiently infects non-adherent hepatoma cells via human sodium taurocholate cotransporting polypeptide. Sci Rep.

[27] Imai S (2013). Epigenetic regulation of organic anion transporting polypeptide 1B3 in cancer cell lines. Pharm Res.

[28] Matsuoka K, Kuranaga Y, Moroi Y (2002). Solubilization of cholesterol and polycyclic aromatic compounds into sodium bile salt micelles (part 2). Biochim Biophys Acta.

[29] Matsuoka K, Maeda M, Moroi Y (2003). Micelle formation of sodium glyco- and taurocholates and sodium glyco- and taurodeoxycholates and solubilization of cholesterol into their micelles. Colloids Surfaces B.

[30] Cohen DE, Angelico M, Carey MC (1990). Structural alterations in lecithin-cholesterol vesicles following interactions with monomeric and micellar bile salts: physical-chemical basis for subselection of biliary lecithin species and aggregative states of biliary lipids during bile formation. J Lipid Res.

[31] Heuman DM, Bajaj RS, Lin Q (1996). Adsorption of mixtures of bile salt taurine conjugates to lecithin-cholesterol membranes: implications for bile salt toxicity and cytoprotection. J Lipid Res.

[32] Mello-Vieira J (2013). Cytotoxic bile acids, but not cytoprotective species, inhibit the ordering effect of cholesterol in model membranes at physiologically active concentrations. Biochim Biophys Acta.

[33] Wang DQ, Carey MC (1996). Complete mapping of crystallization pathways during cholesterol precipitation from model bile: influence of physical-chemical variables of pathophysiologic relevance and identification of a stable liquid crystalline state in cold, dilute and hydrophilic bile salt-containing systems. J Lipid Res.

[34] Copple BL, Li T (2016). Pharmacology of bile acid receptors: Evolution of bile acids from simple detergents to complex signaling molecules. Pharmacol Res.

[35] Puglielli L (1994). Protective role of biliary cholesterol and phospholipid lamellae against bile acid-induced cell damage. Gastroenterology.

[36] Nibbering CP (2001). Regulation of biliary cholesterol secretion is independent of hepatocyte canalicular membrane lipid composition: a study in the diosgenin-fed rat model. J Hepatol.

[37] Lowe PJ, Coleman R (1981). Membrane fluidity and bile salt damage. Biochim Biophys Acta.

[38] Helenius A, Simons K (1975). Solubilization of membranes by detergents. Biochim Biophys Acta.

[39] Palmeira CM, Rolo AP (2004). Mitochondrially-mediated toxicity of bile acids. Toxicology.

[40] Smit JJ (1993). Homozygous disruption of the murine mdr2 P-glycoprotein gene leads to a complete absence of phospholipid from bile and to liver disease. Cell.

[41] Lammert F (2004). Spontaneous cholecysto- and hepatolithiasis in Mdr2−/− mice: a model for low phospholipid-associated cholelithiasis. Hepatology.

[42] Tougeron D, Fotsing G, Barbu V, Beauchant M (2012). ABCB4/MDR3 gene mutations and cholangiocarcinomas. J Hepatol.

[43] Jungst C, Lammert F (2013). Cholestatic liver disease. Dig Dis.

[44] Geuken E (2004). Rapid increase of bile salt secretion is associated with bile duct injury after human liver transplantation. J Hepatol.

[45] Langheim S (2005). ABCG5 and ABCG8 require MDR2 for secretion of cholesterol into bile. J Lipid Res.

[46] Allen RM (2012). miR-33 controls the expression of biliary transporters, and mediates statin- and diet-induced hepatotoxicity. EMBO Mol Med.

[47] Yoshikado T (2011). Itraconazole-induced cholestasis: involvement of the inhibition of bile canalicular phospholipid translocator MDR3/ABCB4. Mol Pharmacol.

[48] Nervi F (1989). Influence of legume intake on biliary lipids and cholesterol saturation in young Chilean men. Identification of a dietary risk factor for cholesterol gallstone formation in a highly prevalent area. Gastroenterology.

[49] Duane WC (1997). Effects of legume consumption on serum cholesterol, biliary lipids, and sterol metabolism in humans. J Lipid Res.

[50] Leiss O, Meyer-Krahmer K, von Bergmann K (1986). Biliary lipid secretion in patients with heterozygous familial hypercholesterolemia and combined hyperlipidemia. Influence of bezafibrate and fenofibrate. J Lipid Res.

[51] Schonewille M (2016). Statins increase hepatic cholesterol synthesis and stimulate fecal cholesterol elimination in mice. J Lipid Res.

[52] Kallien G, Lange K, Stange EF, Scheibner J (1999). The pravastatin-induced decrease of biliary cholesterol secretion is not directly related to an inhibition of cholesterol synthesis in humans. Hepatology.

[53] Gohlke H, Schmitz B, Sommerfeld A, Reinehr R, Haussinger D (2013). alpha5 beta1-integrins are sensors for tauroursodeoxycholic acid in hepatocytes. Hepatology.

[54] Nagahashi M (2016). The roles of bile acids and sphingosine-1-phosphate signaling in the hepatobiliary diseases. J Lipid Res.

[55] Pean N (2013). The receptor TGR5 protects the liver from bile acid overload during liver regeneration in mice. Hepatology.

[56] Studer E (2012). Conjugated bile acids activate the sphingosine-1-phosphate receptor 2 in primary rodent hepatocytes. Hepatology.

[57] Morita SY, Deharu Y, Takata E, Nakano M, Handa T (2008). Cytotoxicity of lipid-free apolipoprotein B. Biochim Biophys Acta.

[58] Morita SY, Ueda K, Kitagawa S (2009). Enzymatic measurement of phosphatidic acid in cultured cells. J Lipid Res.

[59] Hojjati MR, Jiang XC (2006). Rapid, specific, and sensitive measurements of plasma sphingomyelin and phosphatidylcholine. J Lipid Res.

[60] Allain CC, Poon LS, Chan CS, Richmond W, Fu PC (1974). Enzymatic determination of total serum cholesterol. Clin Chem.

