# Supplementary material for: Cholesterol attenuates cytoprotective effects of phosphatidylcholine against bile salts
Source: Sci Rep. 2017 Mar 22;7:306. doi: 10.1038/s41598-017-00476-2 (PMC5428433; doi:10.1038/s41598-017-00476-2)
Supplement: Supplementary file 1 — Supplementary Tables [file 41598_2017_476_MOESM1_ESM.pdf]

# Supplementary Information

## Cholesterol attenuates cytoprotective effects of phosphatidylcholine against bile salts

Yoshito Ikeda, Shin-ya Morita* and Tomohiro Terada

Department of Pharmacy, Shiga University of Medical Science Hospital, Otsu City, Shiga 520-2192, Japan

| | $LD_{50}$ (mM) |
|---|---|
| **HepG2 cells** | |
| NaC | 13.1 |
| NaTC | 12.5 |
| NaGC | 14.2 |
| NaDC | 1.12 |
| NaTDC | 1.24 |
| NaGDC | 1.25 |
| NaCDC | 1.48 |
| NaTCDC | 1.36 |
| NaGCDC | 1.63 |
| **Primary human hepatocytes** | |
| NaDC | 1.16 |
| NaTDC | 1.58 |

**Supplementary Table S1. $LD_{50}$ of bile salts for HepG2 cells and primary human hepatocytes.** $LD_{50}$ values of bile salts were determined by nonlinear regression curve fitting of concentration-response data from LDH release assay (Figs. 1 and 3).

| | IC$_{50}$ of PC (mM) | |
|---|---|---|
| | Bile salt/PC | Bile salt/PC/Cholesterol |
| **HepG2 cells** | | |
| 25 mM NaC | 19.1 | 25.1 |
| 25 mM NaTC | 10.7 | 9.00 |
| 25 mM NaGC | 6.95 | 6.21 |
| 25 mM NaDC | Not definable | Not definable |
| 25 mM NaTDC | 18.1 | 19.0 |
| 25 mM NaGDC | 16.1 | 16.9 |
| 25 mM NaCDC | Not definable | Not definable |
| 25 mM NaTCDC | 16.4 | 18.6 |
| 25 mM NaGCDC | 17.3 | 17.8 |
| 15 mM NaDC | 22.4 | Not definable |
| 15 mM NaCDC | 14.3 | 17.5 |
| **Primary human hepatocytes** | | |
| 25 mM NaDC | 21.5 | 27.7 |
| 25 mM NaTDC | 9.09 | 8.84 |

**Supplementary Table S2. IC$_{50}$ of PC for inhibition of bile salt cytotoxicity to HepG2 cells and primary human hepatocytes.** IC$_{50}$ values of PC were determined by nonlinear regression curve fitting of concentration-response data from LDH release assay (Figs. 2 and 4).
